# Supplementary material for: Temporal Trends in Air Pollution Exposure across Socioeconomic Groups in The Netherlands
Source: Int J Environ Res Public Health. 2024 Jul 26;21(8):976. doi: 10.3390/ijerph21080976 (PMC11353980; doi:10.3390/ijerph21080976)
Supplement: Supplementary file 1 [file ijerph-21-00976-s001.zip › ijerph-3069005-supplementary.pdf]

Supplement File 1: Temporal trends in air pollution exposure across socioeconomic groups in the Netherlands

Table S1a: Differences in average $PM_{2.5}$ concentrations between 2014 and 2019 by socioeconomic position group and urbanicity (2014 to 2019)

Urbanicity[1]	SEP group[2]	$PM_{2.5}$ (µg/m³) – mean 2014	$PM_{2.5}$ (µg/m³) – mean 2019	Absolute difference	Relative difference (%)
**Nationwide**	**1**	13.63	10.28	-3.35	-24.58
	**2**	13.46	10.19	-3.28	-24.33
	**3**	13.31	10.09	-3.22	-24.18
	**4**	13.23	10.05	-3.18	-24.03
	**5**	13.22	10.05	-3.18	-24.02
	**6**	13.21	10.03	-3.18	-24.06
	**7**	13.25	10.07	-3.19	-24.06
	**8**	13.27	10.07	-3.2	-24.13
	**9**	13.33	10.13	-3.2	-23.98
	**10**	13.59	10.31	-3.28	-24.12
**Rural to moderately urbanised**	**1**	13.01	9.83	-3.17	-24.4
	**2**	12.96	9.82	-3.14	-24.24
	**3**	12.9	9.78	-3.12	-24.21
	**4**	12.92	9.8	-3.12	-24.12
	**5**	12.93	9.8	-3.13	-24.18
	**6**	12.94	9.82	-3.13	-24.15
	**7**	12.99	9.84	-3.15	-24.23
	**8**	13.03	9.87	-3.16	-24.26
	**9**	13.08	9.91	-3.17	-24.2
	**10**	13.22	10.01	-3.21	-24.3
**Highly urbanised**	**1**	14.18	10.64	-3.55	-25
	**2**	14.15	10.61	-3.53	-24.98
	**3**	14.09	10.58	-3.51	-24.93
	**4**	14.05	10.55	-3.5	-24.9
	**5**	14.05	10.56	-3.49	-24.84
	**6**	14.03	10.54	-3.49	-24.91
	**7**	14.03	10.56	-3.47	-24.76
	**8**	14.06	10.58	-3.48	-24.75
	**9**	14.1	10.62	-3.47	-24.64
	**10**	14.29	10.78	-3.52	-24.6

[1]Urbanicity: Rural to moderately urbanised areas (< 2000 addresses/km2). Highly urbanised (>2000 addresses/km2)

[2]Socioeconomic position (SEP) group: Decile scores ranging from the lowest SEP (1) to the highest SEP (10)

Table S1b: Differences in average PM_{10} concentrations between 2014 and 2019 by socioeconomic position group and urbanicity (2014 to 2019)

Urbanicity[1]	SEP group[2]	$PM_{2.5}$ (μg/m³) – mean 2014	$PM_{2.5}$ (μg/m³) – mean 2019	Absolute difference	Relative difference (%)
**Nationwide**	**1**	21	17.88	-3.12	-14.86
	**2**	20.79	17.74	-3.06	-14.7
	**3**	20.6	17.58	-3.02	-14.67
	**4**	20.51	17.52	-3	-14.6
	**5**	20.5	17.51	-2.99	-14.57
	**6**	20.48	17.49	-2.99	-14.58
	**7**	20.53	17.55	-2.98	-14.52
	**8**	20.56	17.55	-3.01	-14.65
	**9**	20.63	17.64	-2.99	-14.47
	**10**	20.93	17.92	-3.01	-14.38
**Rural to moderately urbanised**	**1**	20.22	17.08	-3.14	-15.51
	**2**	20.16	17.08	-3.08	-15.28
	**3**	20.1	17.03	-3.07	-15.28
	**4**	20.12	17.06	-3.06	-15.21
	**5**	20.14	17.07	-3.06	-15.22
	**6**	20.15	17.1	-3.05	-15.14
	**7**	20.21	17.14	-3.07	-15.18
	**8**	20.27	17.18	-3.09	-15.24
	**9**	20.33	17.24	-3.08	-15.16
	**10**	20.47	17.37	-3.1	-15.16
**Highly urbanised**	**1**	21.69	18.52	-3.17	-14.63
	**2**	21.64	18.48	-3.16	-14.61
	**3**	21.56	18.43	-3.13	-14.52
	**4**	21.51	18.41	-3.1	-14.4
	**5**	21.5	18.42	-3.08	-14.32
	**6**	21.47	18.41	-3.07	-14.29
	**7**	21.48	18.45	-3.02	-14.08
	**8**	21.51	18.48	-3.03	-14.08
	**9**	21.56	18.54	-3.02	-14.01
	**10**	21.81	18.77	-3.04	-13.94

[1]Urbanicity: Rural to moderately urbanised areas (< 2000 addresses/km2). Highly urbanised (>2000 addresses/km2)

[2]Socioeconomic position (SEP) group: Decile scores ranging from the lowest SEP (1) to the highest SEP (10)

Table S1c: Differences in average NO_2 concentrations between 2014 and 2019 by socioeconomic position group and urbanicity (2014 to 2019)

Urbanicity[1]	SEP group[2]	$PM_{2.5}$ (μg/m³) – mean 2014	$PM_{2.5}$ (μg/m³) – mean 2019	Absolute difference	Relative difference (%)
**Nationwide**	**1**	22.06	18.97	-3.09	-13.99
	**2**	21.06	18.32	-2.74	-13.01
	**3**	20.25	17.72	-2.53	-12.51
	**4**	19.71	17.36	-2.35	-11.9
	**5**	19.62	17.31	-2.3	-11.75
	**6**	19.57	17.23	-2.34	-11.97
	**7**	19.73	17.41	-2.32	-11.78
	**8**	19.62	17.28	-2.35	-11.96
	**9**	19.89	17.64	-2.25	-11.29
	**10**	21.15	18.59	-2.56	-12.11
**Rural to moderately urbanised**	**1**	18.46	16.05	-2.41	-13.06
	**2**	18.22	15.93	-2.3	-12.61
	**3**	17.99	15.71	-2.28	-12.67
	**4**	17.92	15.7	-2.22	-12.39
	**5**	17.96	15.71	-2.25	-12.53
	**6**	18.05	15.78	-2.27	-12.55
	**7**	18.19	15.89	-2.31	-12.68
	**8**	18.2	15.9	-2.3	-12.66
	**9**	18.39	16.14	-2.26	-12.28
	**10**	18.97	16.52	-2.44	-12.89
**Highly urbanised**	**1**	25.23	21.3	-3.93	-15.57
	**2**	24.89	21.05	-3.84	-15.43
	**3**	24.54	20.82	-3.72	-15.17
	**4**	24.31	20.64	-3.66	-15.06
	**5**	24.26	20.66	-3.6	-14.84
	**6**	24.2	20.58	-3.61	-14.94
	**7**	24.27	20.77	-3.5	-14.42
	**8**	24.3	20.81	-3.5	-14.38
	**9**	24.43	21	-3.43	-14.05
	**10**	25.25	21.71	-3.54	-14.03

[1]Urbanicity: Rural to moderately urbanised areas (< 2000 addresses/km2). Highly urbanised (>2000 addresses/km2)

[2]Socioeconomic position (SEP) group: Decile scores ranging from the lowest SEP (1) to the highest SEP (10)
